# Health care providers’ and mothers’ perceptions about the medicalization of female genital mutilation or cutting in Egypt: a cross-sectional qualitative study

**DOI:** 10.1186/s12914-019-0202-x

**Published:** 2019-08-27

**Authors:** Omaima El-Gibaly, Mirette Aziz, Salma Abou Hussein

**Affiliations:** 1 Assiut University, Assiut, Egypt; 2 Population Council, Cairo, Egypt

**Keywords:** Female genital mutilation/cutting, Medicalization, Egypt, Gender equity, Sexual and reproductive health rights

## Abstract

**Background:**

Female genital mutilation/cutting (FGM/C) is a traditional harmful practice that has been prevalent in Egypt for many years. The medicalization of FGM/C has been increasing significantly in Egypt making it the country with the highest rate of medicalization. In this qualitative study, we explored the drivers and motives behind why healthcare professionals perform FGM/C and why mothers rely on them to perform the practice on their daughters.

**Methods:**

The study drew on a “mystery client” approach, coupled with in-depth interviews (IDIs) and focus group discussions (FGDs) with health care providers (i.e. physicians and nurses) and mothers. It was conducted in three geographic areas in Egypt: Cairo, Assiut and Al Gharbeya.

**Results:**

Study findings suggest that parents who seek medicalized cutting often do so to minimize health risks while conforming to social expectations. Thus, the factors that support FGM/C overlap with the factors that support medicalization. For many mothers and healthcare providers, adherence to community customs and traditions was the most important motive to practice FGM/C. Also, the social construction of girls’ well-being and bodily beauty makes FGM/C a perceived necessity which lays the ground for stigmatization against uncut girls. Finally, the language around FGM/C is being reframed by many healthcare providers as a cosmetic surgery. Such reframing may be one way for providers to overcome the law against FGM/C and market the operation to the clients.

**Conclusion:**

These contradictions and contestations highlighted in this study among mothers and healthcare providers suggest that legal, moral and social norms that underpin FGM/C practice are not harmonized and would thus lead to a further rise in the medicalization of FGM/C. This also highlights the critical role that health providers can play in efforts to drive the abandonment of FGM/C in Egypt.

**Electronic supplementary material:**

The online version of this article (10.1186/s12914-019-0202-x) contains supplementary material, which is available to authorized users.

## Background

Approximately 200 million women and girls in 30 countries have undergone female genital mutilation/ cutting (FGM/C) [1]. FGM/C is defined as “all procedures involving partial or total removal of the external female genitalia or other injury to the female genital organs for non-medical reasons” [2]. Most women and girls who are cut live in Africa and Asia. Universally considered a violation of human rights, FGM/C not only physically harms women and girls, but it also causes psychological problems because of the traumatic experiences the victims undergo.

To reduce the incidence of these complications whilst complying with a cultural demand, and as a response to the emphasis on the health risks characterized in anti-FGM/C campaigns, FGM/C is increasingly carried out by health care providers “medicalization”. The World Health Organization (WHO) defines medicalization as “the situation in which FGM/C is practiced by any category of healthcare provider, whether in a public or a private clinic, at home or elsewhere” [3]. These medical health professionals may include physicians, nurses and/or midwives. Demographic and Health Surveys (DHS) data shows that medicalization has increased particularly in Egypt, Sudan, Kenya, Nigeria, Guinea, Yemen and, more recently, in Indonesia. In many of these countries at least one-third of women reported that their daughters were cut by a trained healthcare provider [4–6]. Further, a higher number of younger women compared with older women have been cut by medical personnel, demonstrating a trend toward medicalization [7].

With this rising trend, understanding why healthcare providers perform the practice and why women/families rely on them has become essential. In an integrative review of the literature on the motivations behind health practitioners performing FGM/C in many practicing countries as well as host countries [8], the main reasons highlighted were; (1) the belief that having a health practitioner performing FGM/C on a girl would be less harmful than being performed by a traditional birth attendant (i.e. harm reduction); (2) the belief that FGM/C is a cultural practice; (3) the financial gains of performing the practice and (4) feeling pressured by the community to perform the practice. Such reasons may vary, however, from one country to the other.

Looking at Egypt, FGM/C is a deeply rooted practice, where the prevalence of FGM/C among women and girls ages 15 to 49 years old was 87% in 2015 [7]). However, daughters of younger ages are less likely to be cut; 68% among girls ages 18–19 years [9]. Regarding age, traditionally girls are cut slightly before or at puberty [9, 10].

Although the practice has become illegal in 2008 and rates are declining, Egypt continues to witness a drastic surge in FGM/C medicalization over the past 20 years, making it the country with the highest rate of medicalization. According to Egypt Demographic and Health Survey (EDHS) data, the rate of medicalization among girls and young women ages 19 years and younger rose from 55% in 1995 [11] to 74% in 2014 [9]. This increase could be attributed to a 1994 medical decree issued by MoHP that allowed FGM/C to be performed only by physicians in designated facilities at fixed times and cost [12]. The decree was initially issued with the intention of reducing complications and, eventually, ending the practice. However, the subsequent deaths of girls who were excised in hospitals pressured the ministry to reissue a ban on the practice in all hospitals.

With increasing awareness of the adverse health consequences and greater access to health care services, healthcare providers have become increasingly involved in performing FGM/C. Campaigns to abandon FGM/C in Egypt gained momentum in the 1990s where many of the messages conveyed and primarily highlighted the immediate physical harms of FGM/C which may have partly contributed to the rising medicalization of FGM/C. Also, according to a study conducted in Qalyubeya [13], mothers chose for their daughters to be cut by a health care provider to mitigate risks. The main reason given for having daughters cut by health care providers rather than dayas (traditional birth attendants) was that medical professionals had better training and knowledge about performing FGM/C and, consequently, girls faced lower risks of health complications. Mothers also reported that they sought physicians’ opinion on whether their daughters needed FGM/C and, in some cases, physicians recommended cutting [13]. Similarly, a study on medicalization in Egypt found that 51% of physicians who performed FGM/C operations reportedly did so out of conviction and belief, while 30% did so for profit. The rest claimed the reason was harm reduction by preventing parents from sending their daughters to dayas [14]. Thus, physicians may not merely respond to patients’ demands, but may believe that FGM/C is medically indicated for some girls (WHO 2010). The trust and credibility conferred in them, because of their profession, has concerning implications for the prospects of abandonment.

Another study examined medical students’ attitudes and knowledge about FGM/C and found that only 31% had a good level of knowledge. Fifty-nine percent of students favored discontinuation, with a greater proportion of females than males favoring discontinuation (76% versus 39%). The study found that men, who lived in Upper Egypt, and who were of rural origin, and women who were cut were more likely to support FGM/C compared to women from Lower Egypt, of urban origin, and uncut women, respectively. Students in pre-clinical studies were also more likely to support FGM/C than those in medical study [15]. The low levels of knowledge among medical students suggests a need to understand and analyze the kinds of information they receive during their training and studies in medical school.

Although medicalization of FGM/C has been well-documented in Egypt, a deeper understanding of the drivers of medicalization is needed to inform the conceptualization and development of appropriate interventions geared toward the abandonment of the practice. Little is known about healthcare providers’ (i.e., physicians and nurses) beliefs and motivations around the practice, and about the demand for medicalized FGM/C. To address this need, this study sought to identify the factors that support the continuation or questioning of FGM/C and the reliance on health care providers to perform FGM/C.

## Methods

We conducted a qualitative study among healthcare providers and mothers to identify the drivers and motives behind the shift in the medicalization of FGM/C. The study drew on a “mystery client” approach, coupled with in-depth interviews (IDIs) and focus group discussions (FGDs) with health care providers (i.e. physicians and nurses) and mothers.

### Study sites and population

To capture possible geographic variability, data were collected in three governorates: Assiut, Cairo, and Al Gharbeya. Cairo and Al Gharbeya are governorates in Lower Egypt, while Assiut is a governorate in Upper Egypt. Populations in these governorates have different socio-economic and socio-demographic characteristics as well as variable customs and traditions. Levels of medicalization of FGM/C are also variable. In 2014, about 39% of ever-married cut women and girls ages 15 to 49 in Lower Egypt were cut by a health care provider, compared with 34% in Upper Egypt [9].

The target population comprised physicians (males and females), nurses and mothers of cut daughters. We aimed to recruit physicians and nurses comprising a mix of rural and urban-based practitioners, and young/older physicians with different expertise levels. A total of 100 physicians and 36 nurses participated in the study. Physicians were general surgeons, gynecologists or general practitioners. All nurses, by default, are women in Egypt. Health care providers with these criteria (physicians and nurses) were recruited by health directorates in the three governorates via invitations sent to different primary health care units, district and general hospitals.

Thirty mystery client visits were carried out; 20 visits in private clinics in Assiut (*n* = 10) and AlGharbeya (n = 10) and in NGOs clinics in Cairo (n = 10). The selected physicians were either general surgeons or gynecologists. Key informants; community health workers or nurses identified the popular physicians in the communities to be visited. All were selected from a larger pool of physicians who signed an informed consent of accepting being visited by a “mystery client” sent by the governorates health directorates to assess quality of reproductive health counseling for research purpose only, with ensured confidentiality. In Assiut and AlGharbeya, five of the selected physicians were known by the key informants to perform FGM/C while the other five were randomly selected surgeons and/or obstetricians/gynecologists. In Cairo, visits were only made to public clinics run by NGOs because of difficulties obtaining consent from physicians working in private clinics. The timing of the visit was not disclosed.

The study included 39 women from the three governorates: 18 from Assiut, 11 from Al Gharbeya and 10 from Cairo. Mothers were eligible to participate if they had a daughter aged 14 years or younger who had been cut within the two years preceding data collection. They were recruited by NGOs personnel working at the grass root level in the study sites.

### Data collection

Data were collected between December 2016 and February 2017 using three different methods:

#### Mystery client visits

Mystery client visits were used to understand physicians’ responses towards mothers who were seeking to have their daughters cut and those who were ambivalent about FGM/C and were seeking advice. Four research assistants were trained to present anonymously at each physician’s clinic and to act out two scenarios. One scenario involved an undecided mother who wanted to cut her 18-year-old daughter on her daughter’s fiancé’s request but was afraid of its complications. The other involved a mother who was determined to cut her 12-year-old daughter and visited the physician to set an appointment for the procedure and asked about technicalities/logistics (i.e., timing, venue and cost). These scenarios were constructed based on the findings of the performed FGDs with physicians who mentioned that they are usually asked to cut girls at the age of 10–12 or asked to cut older girls about to get married. Mothers also in the FGDs mentioned that they seek the consultation of physicians either for cutting their girls at the age of puberty or when asked by their daughter’s fiancée or his family to cut the bride as a prerequisite for marriage. Both scenarios were discussed and agreed upon by the advisory committee of the National Population Council in Egypt to explore the responses of physicians when they face both situations.

The mystery clients were trained to ask questions related to the conditions that necessitated FGM/C, the advantages and disadvantages of FGM/C, complications of FGM/C, consequences on marital sexual relations, the immediate risks of the operation, the cost, any precautions prior to the encounter, place of performing the procedure, using anesthesia and healing time.

The research assistants visited the clinics in pairs. During the visit one of them acted as a mother seeking consultation on FGM/C and the other acted as an accompanying relative. Appointments were made ahead of the visits via telephone. The research assistants documented the discussion that had taken place with the physician immediately after the visit. The debrief included the physician’s response to the request to perform FGM/C; his or her opinion about the importance of FGM practice and whether FGM/C was indicated or not; the physician’s beliefs about the most suitable age for performing FGM/C; feedback on the effects of FGM/C on marital sexual relations; discussions on the advantages, disadvantages and expected complications of FGM/C, place and time of conducting the operation, using anesthesia and the cost of the operation.

#### Focus group discussions

Four focus group discussions (FGDs) were performed in each governorate: three FGDs with health care providers (male and female physicians, and nurses) and one FGD with mothers. FGDs with health care providers were designed to explore their attitudes regarding FGM/C, medicalization of FGM/C, gender norms, perceptions of female sexuality and its links to FGM/C, as well as their level of preparedness to consult couples on sexual health issues (i.e. sexual health knowledge, gender issues related to rights of men and women, etc.). FGDs with mothers of cut girls aimed to understand their motivations for FGM/C and their choice of health care provider. FGDs also explored the mothers’ understanding of the messages against FGM/C and the role of their social network on making the decision to cut their girls.

Each FGD lasted between 90 and 120 min. Discussions were guided by a semi-structured discussion guide that was designed for this study and were audio-recorded [16] (Additional file 1). The FGDs were transcribed by note takers while preserving the anonymity of the participants.

#### In depth interviews

Six in-depth interviews (IDIs) were conducted with physicians. In each governorate, one male and one female physician were interviewed. The interviews explored sensitive issues related to female and male sexual health problems and sexuality and FGM/C in medical practice. Interviews were guided by a semi-structured interview guide that was designed for this study [16] (Additional file 1). All IDIs were audiotaped and lasted between 60 and 90 min.

#### Ethical considerations

Ethical approvals were obtained from the Ministry of Health and Population, the Institutional Review Board (IRB) of Assiut University, and the Population Council’s IRB. The letter of approval from the Ministry of Health and Population clearly stated that the identities of the health providers would not be shared with the Ministry or any other entity/individual. The Ministry was responsible for informing physicians that there was a study assessing the deficits of providing reproductive health counseling to mothers/parents. Forty physicians in Assiut and Al Gharbeya governorates and physicians of two NGOs’ public clinics in Cairo consented in writing to the mystery client visits. Physicians were informed during the consenting process that their clinics may or may not be sampled. However, the exact timing of the study/visit was not disclosed. The confidentiality of the performed visits was protected by replacing the names of physicians by numbers and obscuring the location of the visited clinics in the transcripts. Any personal information which might disclose the identity of the participants was not inserted in the manuscripts.

FGD participants were informed about the objective of the study and how their confidentiality would be protected. All participants were assured that their participation in the study was completely voluntary and that they could withdraw at any point in the study. Written informed consent and permission for digital recording were sought. Confidentiality of the participants was preserved by removing all names from the transcribed files and using numbers or different names. The recorded digital tapes were kept only with the researcher.

#### Analysis

Audio files and notes from the IDIs, FGDs and mystery client visits were transcribed in Arabic. Data analysis was performed using the inductive thematic analysis methodology [17]). The transcripts were read repeatedly, and the raw data were coded thematically. Codes and labels were attached to portions of the text related to a specific theme, leading to a set of descriptive themes and sub-themes for each transcript. Data units were constantly compared to identify similarities and variations within categories. All codes were then clustered into themes and sub-themes. The themes were confirmed, modified or discarded from the ongoing analysis by re-examinations of earlier data and considerations of subsequent data collection.

## Results

### FGM/C is perceived to be entrenched in custom and tradition

As expected in the study context, respondents considered FGM/C an important cultural obligation. Across the board, the compulsion to adhere to community customs and traditions was revealed to be mothers’, particularly those who were older, most important motivation for cutting their daughters. Findings from the IDIs and FGDs suggest that mothers see FGM/C as an essential practice and therefore end up cutting their daughters without critically assessing their decision to do so. Pressure from female family members and other community members may further reinforce the practice. These views are highlighted in the following quotes;

“*We grew up finding our grandmothers, mothers and all people circumcising; [it is] a tradition that you have to do.”* Mother, Cairo

*“Circumcision has no advantages, but we grew up and found our folks that way.*” Mother, Cairo

“*I circumcised my daughters [so] that my conscience would be in peace, to be like all other people.”* Nurse, Assiut

As further testament to the extent of social pressure faced by mothers, many participants were not particular about the type or extent of the cut. They accepted the removal of just a tiny part of the external genitalia for their daughters to be considered “cut” as illustrated in the following quote;

“*I brought the doctor to my home and he said ‘no need’, so I replied saying ‘just for the people here, take a tiny scrap for the talks.”* Nurse, Assiut

The mystery client exercise unearthed similar findings, as demonstrated by this quote from a male physician in Al Gharbeya;

“*We cut her. What’s the problem? There are no problems from circumcision now, bring your daughter today and if needed I will cut her immediately tomorrow. We can cut a small part just for her mother-in-law.”*

Narratives from study participants suggest that health practitioners are not immune to the societal pressure to perform FGM/C on their clients. This pressure is brought about by the perceived entrenchment of this customary practice. In rural areas in particular, refusal to perform FGM/C by health providers (especially recent graduates) was reported to pose reputational risk and was said to result in a loss of community trust. Physicians who refuse to perform FGM/C were reported to have lower client loads and, therefore, reduced income. As a male physician in Cairo explained;

“*Doctors in the villages practice a lot (of circumcision) because if he does not do it, the people will be upset with him and no one will come to him, even for a cough. He will be marked.”*

Beyond reputational risk, health practitioners pointed out that they were also members of the communities in which they practiced. This reality posed challenges for maintaining professional distance from the customary entrenchment of FGM/C. Despite having a medical education, physicians themselves, especially those who were from rural areas, were also affected by the prevailing cultural norms and beliefs of their community. The pressure to conform to cultural norms sometimes meant that they performed FGM/C although they were aware about the medical risks involved. The following quotes from health providers illustrate these points;

“*Doctors are part of the people, part of society and people who are convinced do it in particular because we didn’t come across it in our medical education.”* Male physician, Cairo

“*When my daughter was 12, I know that this is not good, but I have fears for her and people would talk [gossip]. I refused to inspect her, so I told her I am going to take you to a colleague of mine just to have a look. She was crying all the way from home until we went to the doctor and asked me, “Mama, what are the advantages of circumcision?” Look, I am going to tell you something. Sometimes you know that this thing is wrong, but you do it out of fear. Do you understand?”* Female physician, Cairo

### Performing FGM/C has financial benefits for providers

According to some physicians, financial benefits were the most important motivator for physicians to perform FGM/C. The narratives from physicians suggested that those who performed the practice due to the financial benefit may understand its hazards and illegality, but still perform it in secrecy. These views are articulated in the following quotations;

“*More than 50 percent of the doctors in the rural areas do these things for several reasons. First for financial gains and trust of people that he [the doctor] responded to their needs and they will come to him for other matters. And if he does not do it he will be stigmatized.”* Male physician, Cairo

“*Believe me, FGM/C in Egypt is not because people ask for it, but it is driven by physicians and nurses. I swear if it wasn’t for the financial benefits out of it, no girl would have been circumcised in Egypt. People get easily convinced by health care providers.”* Female physician, Al Gharbeya

When mystery clients asked about the cost and the charges for FGM/C they were given varying amounts ranging from 250 Egyptian Pounds (LE) (US$14) to 1500 LE (US$85). The requested charges were higher for older girls. Physicians noted that they ask for high prices because of the cost of the anesthesia and hospital fees, when performed in a private hospital. As illustrated in the following quote from a male physician in Al Gharbeya, the costs were also reportedly affected by how extensive the procedure was, “*When I see her (examine her) and I know how much I will remove, I will tell you how much I will take”*.

### Some providers view the performance of FGM/C as their religious duty

Providers noted that in some cases, providers’ religious convictions around FGM/C influenced their decision to perform FGM/C. They noted that, in some cases, physicians who are convinced about its importance may perform it for women during delivery, even without being requested to do so.

“*There are people who do it (FGM/C) and are strongly convinced about it, people who are (I don’t want to classify them) Muslim Brotherhood, Sunni, especially the Sunnis, those with beards, and those religiously fanatic, these ones do it out of religious conviction, even if they do not take money. The one who does it for money can cut ten people and the one who does it for religion, can cut a hundred.”* Male physician, Al Gharbeya

Some physicians in Assiut and Al Gharbeya believed that performing FGM/C was a religious obligation despite being condemned by Al-Azhar (Highest Muslim Religious Authority). Other physicians considered FGM/C as “Sunna” – that is, not mandatory, while others refuted a religious basis for FGM/C. Physicians based in Assiut and Al Gharbeya were more likely to believe that there were religious indications for performing FGM/C than physicians based in the other study sites, which may reflect the more conservative culture in Upper Egypt.

“*If it [circumcision] were forbidden, why was it done originally? Okay, why was it done in many Islamic countries? If it was forbidden and haram [religiously wrong], why was it done from the time of my grandmother and your grandmother before us?”* Female physician, Al Gharbeya

As illustrated by the following quotes, some physicians and nurses also believed that “Sunna circumcision” is of the first degree and did not have any negative consequences on marital sexual relations.

“*I may perform FGM/C when needed for the girl, and to avoid the problems which would happen if she was not cut. The Muslim Prophet, when asked about female circumcision, said shorten but don’t over-excise, which means performing a minor cut for the cosmetic appearance.”* Female physician, Al Gharbeya

“*It is okay to cut a small part, just for beautifying the clitoris, and the Prophet said shorten but don’t over-excise, which means that we shouldn’t cut much.*” Nurse, Assiut

### FGM/C is not included in the medical training of health care providers

Despite the high prevalence of FGM/C in Egypt, physicians indicated that FGM/C did not feature in their medical school curriculum or training. Almost all of them mentioned that they only studied the anatomy of the female genital system. Only a few physicians stated they had some exposure to the prevalence of FGM/C in Egypt in the public health curriculum or the reproductive health postgraduate curriculum. As intimated by a male physician in Assiut, *“I never took anything deep about FGM/C. We took the anatomy of the female system. That is the information I have”*.

Given the lack of medical training for performing FGM/C, providers who perform it mentioned that they learned about the practice from their colleagues. They also mentioned that physicians used different techniques because they had no reference. As a male physician in Assiut noted, “*Nothing in medicine taught us how to [perform FGM/C], and I didn’t study it during my years of education. Whether cutting from the right or from the left, it is personal”*.

Most physicians and nurses further indicated that except for training on the anatomy and physiology of the female genital organs, they had received no training on sexual health. Their knowledge about sexual health was limited to what they had learned from self-reading and internet searches. These views are illustrated in the following quotes from health care providers;

*“We had obstetrics/gynecology in the fourth year of medical school and it was one lecture on sex and that year this lecture was removed for political reasons.”* Male physician, Assiut

“*We took physiology and histology but not sexuality. In Assiut University, they talked about the science of orgasm and functions but not sexuality.”* Male physician, Assiut

“*What I know about sexual health comes from the time I was in secondary school when the ‘Always’ (sanitary pads) company came and gave us a brochure on periods, the body, the monthly ova and drawing of the uterus. That is it. I still have a copy [of the brochure].”* Nurse, Al Gharbeya

Few physicians had correct knowledge that sexual health is a broad term encompassing physical and psychosexual aspects, while most providers mentioned incorrect definitions of sexual health. Most of them stated that sexual health only encompasses “sexual intercourse”. Unsurprisingly, therefore, some physicians referred erroneously to the presence of smegma between labia minora and labia majora as an indication for FGM/C (i.e., cutting the labia minora), as it could be a predisposing factor for cancer. In line with this gap in sexual health training, physicians felt ill-equipped to provide counseling on sexuality and sexual health despite the admitted demand for it from their patients. Physicians spoke of their experiences handling clients with sexual health problems. Some stated that they were too embarrassed to discuss sexual health issues with their clients, mentioning that they usually used medical management and failed to provide any counseling.

“*We are not prepared at all. Really, we are asked a lot and we are being exposed to many situations as gynecologists. For example, patients with sexual dysfunction come and say this happens with me and this does not. I am not prepared to respond but it is a basic part of our practice.”* Male physician, Assiut

“*If a newly married woman came seeking my consultation, I can’t tell her anything, not because of embarrassment, just because I don’t know, and I tell her to ask someone else better than me.”* Female physician, Assiut

“*Sexual health? I don’t know what that means. We treat medical issues, but we don’t know how to do sexual health consultations.”* Female physician, Assiut

“*Sexual health consultation is a doctor’s own effort. None of us learned it. Everyone learns by himself. Patients come to us to ask us about sexual health. I tell them, you tell me.”* Female physician, Al Gharbeya

Some physicians also felt that providing sexual health counseling could be unacceptable to the community, especially when provided by an unmarried female physician.

“*There is no sexual education and the society disapproves of that, and what we know is from our own personal effort, but we didn’t learn it [in medical school].”* Female physician, Assiut

“*If she is single [a virgin], even if she is a doctor, they will not accept her consultation. In other words, doctor [X] will be accepted but I won’t be. They would say it is “disgraceful”, it is unacceptable for a girl to talk about sexual health, even if she is a doctor.”* Female physician, Assiut

This gap in training and knowledge arguably helps sustain the practice of FGM/C. If providers do not recognize sexual health as a fundamental part of overall health, and if they do not make the possible connections between poor sexual health and FGM/C, then they are less likely to work towards the abandonment of the practice.

Mothers, on the other hand, are unaware of health care providers’ lack of knowledge and training on FGM/C and underscored their preference to have their daughter cut by health providers rather than dayas. They stated that health providers were a trusted source of health care and highlighted the clean environment in health facilities and the use of sterile equipment. Mothers also reported that health care providers were better trained, cut less tissue, used anesthesia and followed up with patients. Having the procedure performed by trained practitioners, and preferably in a clinic or a hospital, was thought to minimize the health risks, pain, and, even, marital sexual problems, while sustaining the practice to meet cultural norms.

“*The doctor has experience and has been educated. A daya is fine but education is good, a daya would spray an anesthetic but a doctor gives an injection of anesthesia in the side and the girl feels nothing but can see.”* Mother, Cairo

“*Women who have problems with their husbands because they were circumcised by a daya. If it were a doctor, it would be different. I have been circumcised by a doctor. My sisters by a daya. They have problems and I don’t.”* Mother, Assiut

### Social construction of girls’ well-being makes FGM/C a perceived necessity

Further analysis of respondents’ narratives suggests that the practice of FGM/C is driven by the social construction of girls’ well-being in the study setting. Participants’ narratives reveal various concerns that mothers have for their daughters’ health and happiness, and comfort in Egyptian society. The narratives demonstrate that girls’ well-being is constructed around how they are viewed by others in the community. Attributes such as propriety and bodily beauty afford a girl respect from the community. These attributes also increase a girl’s marriageability, particularly in the rural areas. A lack of such characteristics is associated with being uncut a status that is stigmatized. Understandably, therefore, mothers have a shared goal of ensuring their daughters meet community standards (and therefore undergo FGM/C), thus attaining better life chances in general.

Sexual purity, for instance, was a key construct that participants referred to in their conceptualization of girls’ well-being. When referred to, sexual purity for girls was often framed in terms of low (or a total lack of) libido, which was also viewed as evidence of ‘good behavior.’ Respondents in Cairo and Al Gharbeya were more likely than their Assiut peers to express these opinions. These views are illustrated in the following quotes from mothers;

“*She would have more sexual desire than her husband if left uncircumcised that is why they say circumcision is good for girls.*” Mother, Assiut

“*In villages, they say the uncircumcised girl would have much more sexual desire, would like to talk to men all the time instead of girls and would play mainly with boys. So, her behavior would not be good.”* Mother, Assiut

*“I had a daughter her body was* ‘*hot*’ *[sexually excited]. When I circumcised her, she calmed down. I used to tell her you are agitated, but she became good and came back to her mind when I circumcised her. My mother-in-law told me to circumcise her. I sent her to Fayoum because my sister-in-law is a nurse at a doctor*’*s clinic and she circumcised her.*” Mother, Cairo

Some mothers, physicians, and nurses disputed any associations between FGM/C and girls’ sexual excitement and behavior, however, contending that sexual arousal is a complex biological and neurological process, or pointing out the effect of environmental and relational factors on sexual behavior.

Interviews and discussions with study participants demonstrate that girls’ well-being is also linked to their bodily beauty in the study context. Indeed, most mothers in Assiut, for example, did not link FGM/C to sexual behavior, but rather to genital hygiene and cosmoses. Most of them reportedly cut their girls to ensure that they did not have a protruded clitoris that would be erect during sexual intercourse, which they considered unacceptable. Uncut girls were considered to have similar organs to men, which was considered unfeminine and unattractive. These perceptions are illustrated in the following quotes;

“*They say a girl if left uncircumcised would be like a man. We have to cut these things. How can a girl be like a man? If she would not be circumcised, she would be like her husband. This is not right. At the same time, it is cleanliness for her too.*” Mother, Assiut

“*There was a doctor and I heard him saying* “*I was assisting a woman in her delivery and her genitalia looked so ugly from beneath. If I am a doctor and not her husband and see it this way, what does her husband do with her?*” *And I heard the same doctor saying we need to set up a committee of obstetricians/gynaecologists and Al-Azhar to do a medical examination for the girl to decide whether she needs it or not.*” Nurse, Al Gharbeya

“*I saw a girl who has large things, skin protruding down and long and the whole thing looks very bad. I saw many cases like that. I work in rural areas. If you are working in the urban areas, you have not seen anything. I stayed 20 years in rural areas and saw a lot of cases that had to be done [circumcised].*” Male physician, Assiut

The mystery client exercise reinforced these findings, as female and male providers alike repeatedly referred to ‘beauty’ and ‘ugliness’ in regard to girls’ genitalia. As demonstrated below, their words were usually set in the context of assistance and support to ensure the best outcome for the girls concerned within marriage and otherwise:

“*Yeah, there are girls God created them looking normal and beautiful from underneath [genitalia], and there are girls who have large organs. As the prophet said, don*’*t overdo it and cut a little, from the clitoris; we cut a small part because if it [protruding genitalia] is long, it causes problems and looks very ugly.*” Female physician, Al Gharbeya

“*Bring her. I*’*ll take a look (examine her) and then I*’*ll decide. If it [genitalia] looks ugly and will affect marriage, we cut it and if it is okay, we leave it. By the way, there are married women who come for us to do corrections for them. I just have to see what is there. I haven*’*t seen anything...you are telling me something vague. I have to see with my own eyes and will do what is needed for her.*” Male physician, Cairo

Nurses in Al Gharbeya were more likely to support the cosmetic and hygienic indications of FGM/C than providers in other settings. Providers’ focus on enhancing bodily beauty is arguably linked to issues that are perceived to ensure girls’ well-being, including their marriageability. In some rural areas, uncut girls were reportedly required by their fiancés to undergo FGM/C. Moreover, some mothers mentioned that husbands would force uncut wives to get cut to avoid shame or to ensure their wives’ fidelity. The views are illustrated in the quotes below;

“*A man from Upper Egypt married a woman from Lower Egypt and he swore not to consummate the marriage unless she is circumcised.*” Nurse, Assiut

“*People would repeatedly tell him* ‘*your wife is uncircumcised*’*. The whole village knows each other and knows who is and who isn*’*t circumcised.*” Mother, Assiut

### The discursive (re)framing of FGM/C by the medical community casts the practice in a positive light

Findings demonstrate how the language around FGM/C is being reframed by health care providers. As shown in the following quotes, many physicians who performed FGM/C denied that they practiced FGM/C, preferring to refer to the procedure as ‘a cosmetic operation’ instead. It is plausible that providers rely on such terms to free themselves of the blame directed to the physicians when cutting girls. Providers claimed that in most cases, they only cut the labia minora and not the clitoris, except when the clitoris was enlarged and protruded out of the labia minora.

“*A doctor in Assiut in a training workshop said we beautify women, although he has a high scientific degree and knows this practice is harmful.*” Female physician, Assiut

“*I don*’*t call it circumcision, I call it* “*refinement*”*. For me, as a doctor, I don*’*t do this case as female circumcision, I do it as a technical case. For example, after the age of 16 to 17, when everything is clear and there are problems from it, so I do this refinement or cosmetic operation.*” Male physician, Al Gharbeya

“*There is a woman doctor who told me that many people come to ask for this operation. They say they feel that the labia minora is large and she does it to them, but she does not come close to the clitoris. I told her that is circumcision, she said no. People ask for it as a cosmetic need, she considers it cosmetic and not circumcision because it is the labia minora only.*” Female physician, Assiut

“*This is not considered circumcision with the common meaning that we remove the clitoris, but you are beautifying the labia. It*’*s normal. I have patients who are married and do it after marriage and birth too.*” Female physician, Al Gharbeya

In speaking about FGM/C, providers described female genitalia as either ‘normal’ or ‘abnormal,’ depending on the extent of labial protrusion. Provider narratives cast labial protrusion in a negative light. Most physicians and nurses mentioned that they had female patients with oversized genitalia, which they thought should be removed. They mentioned that they examined girls’ genitalia and classified them into “indicated” and “non-indicated” cases for cosmetic correction to the normal shape, which they considered ‘non-protrusion of the clitoris out of the labia minora’. They mentioned several complications of non-removal, such as repeated infection, bad odors, bleeding, sexual excitement and dyspareunia. Some of them considered these cases to be “congenital anomalies” thus providing further rationale for performing FGM/C.

“*There are cases that are in need [of cutting]. If I have an extra finger, I will accept it but others might not especially those who live with me. We don*’*t remove it completely. We make it normal, the extras [skin] are not normal. If someone is going to do something [FGM/C], he has to know, understand and then decide that this has to be removed.*” Male physician, Assiut

“*If anatomically the girl is all right, there is no problem. But if she is not, she should be circumcised. Otherwise, if she does not observe hygiene, she can get a fungal infection. She can also bleed from the extreme friction, and it could cause her pain in sexual intercourse and result in divorce. It has happened in our village that people got divorced because of this issue.*” Male physician, Al Gharbeya

“*Look, the normal size is that when the labia majora are closed. Nothing is protruding from them. That one does not need it but if there are protrusions outside the labia majora, there would be a need [to cut the girl].*” Female physician, Al Gharbeya

### Responses of the physicians to clients’ requests to perform FGM

When physicians were visited in their clinics by the mystery client (an actor mother) and were requested to perform FGM/C to the daughter, they had different responses. Four physicians accepted to perform FGM immediately, and asked the “actor mother” to bring the girl for the operation. They were very supportive to FGM and even convinced the hesitated mothers about its importance regarding the cosmetic and hygienic indications.

Almost half of the physicians (14 out of 30 physicians) asked for examining the girl before taking a decision whether to accept or not to perform such practice. They mentioned that they would decide the need according to the size of the clitoris and labia minora. We can’t conclude what would happen after examination. They could have done so to prepare her for the procedure or to convince the mothers that their girls aren’t in need for such practice, as mentioned by some physicians in the FGDs.

“*I will give you my decision after examining the girl, as circumcision could be indicated for some cases and not for others. Some girls have a large clitoris which needs to be cut. If not, it is not indicated for this case.*” *Male physician, Al Gharbeya*

Six physicians refused to perform FGM but referred the cases to other colleagues who are known to perform such practice. They refused performing FGM either due to being against the practice or because their clinics were not equipped for performing such procedure. Only six physicians were very determined in refusing to perform FGM and also refused to recommend other colleague for performing it.

“*I will be honest with you. I never perform female circumcision, but I will tell you about a trusted colleague who would perform it. This procedure needs to be performed in a private hospital, not a clinic, even if it is more expensive.*” *Female physician, Cairo*

“*I don*’*t circumcise girls. First, this is criminalized by law and the penalty has been also increased recently. Second, it has no indications and has nothing to do with sexual purity. You should better discuss her fianc*é*e and convince him with what I told you.*” *Female physician, Assiut*

## Discussion

After more than 30 years of investments in FGM/C abandonment interventions in Egypt, an alarmingly high number of physicians in Egypt perform FGM/C. This is despite legal sanctions against the practice, being bound by the Hippocratic Oath to do no harm and the declaration of Al-Azhar—the highest religious authority in Egypt—that FGM/C is not part of Islam. Study findings suggest that FGM/C continues to be widely practiced primarily because it ‘protects’ females by limiting their sexual desire and enables them to behave in culturally appropriate ways. However, continued discourse around FGM/C, as well as women’s own experiences, are fueling change to the practice with FGM/C now being performed primarily by health care providers, particularly physicians and nurses, for health reasons. Study findings also suggest that legal, religious, moral and social norms relevant to abandonment of FGM/C practice are not harmonized and result in a mosaic picture of FGM/C practice among the health care workers (physicians and nurses) as well as clients (mothers).

The results show that the drivers of FGM/C practice overlap with drivers of medicalization. The practice of FGM/C among daughters of health care providers, whether nurses or physicians, is an important finding, even if not as common as in the wider community, as it strongly reflects how social norms outweigh the law and medical ethics. This group of providers may perpetuate the practice and drive its continuation. Many of the reported reasons for maintaining the practice by health care providers are consistent with what is reported in the literature: the belief that it is a cultural or religious obligation, harm reduction, physicians perceived by parents (mothers) as offering more safety and better handling of complications, and financial benefit.

Study findings suggest that most nurses and some physicians are still strongly influenced by their own cultural group convictions and report practicing FGM/C in their own families because they still consider it a cultural obligation to which they must adhere. This has been reported by other studies in different African countries [18, 19]. Other than practicing FGM/C in their own families, culture affects the practice of FGM/C by physicians in different ways. First, physicians face social sanctions (loss of respect and trust) if they refuse to cut girls in rural communities with possible financial consequences. For some of them these sanctions have a stronger influence than moral and legal norms for abandonment of FGM/C. Second, some physicians tolerate the practice even if they themselves do not believe it in it and, therefore, are willing to refer parents to practicing providers. This suggests that many providers do not understand that the practice is a criminal act against a child or that FGM/C has consequences on female health and sexuality.

Study findings underscore three issues that should be addressed in FGM/C abandonment efforts: renaming FGM/C as a cosmetic operation and not cutting; justifying a ‘Sunna type’ of FGM/C by health care providers; and the gap in undergraduate medical and nursing education on FGM/C and female sexuality and sexual health. Reframing FGM/C as cosmetic surgery is problematic because it legitimizes the practice. The law against FGM/C in Egypt has been repeatedly modified but has not dealt with female genital cosmetic surgery (FGCS) among young adult females more than 18 years old. FGCS is a global phenomenon that has triggered feminist activism [20]. Although professional bodies, such as the American College of Obstetrics and Gynecology (ACOG) issued a statement in 2007 indicating ‘these procedures were not medically indicated, nor is there documentation of their safety and effectiveness’, it was not decisive on sanctions against surgeons practicing or advertising such surgeries [21]. In a country such as the United States, where the FGM/C socio-cultural context and its medicalization is different, FGCS may not be a significant issue, but in Egypt, with high FGM/C prevalence and widespread medicalization, FGCS can have extensive negative consequences and needs to be addressed by professional and legal bodies. In a context marked by significant gender inequality, young adult females in Egypt may consent to such operations underestimating the long-term consequences on their sexual health. Cosmetic surgery is also an implicit way to market FGM/C to mothers of children.

The framing of FGM/C as ‘Sunna’ is also problematic. The word ‘Sunna’ for Muslims means following the Prophet’s practices. Sunna circumcision for Muslims is a requirement for boys, but not for girls, and it entails removal of the prepuce of the penis. Abandonment activities against FGM/C must clearly state that there is no form of FGM/C that is ‘Sunna type’. Doing so, will ensure the separation of FGM/C from any religious connotations.

### Study implications

With the culture of FGM/C still strong, tackling medicalization would only be possible by addressing both the demand for it by the community, as well as its supply by medical professionals. Sexual education should be included in school curriculum and integrated in social marketing campaigns for FGM/C abandonment. It should tackle not only FGM/C as a practice, but also correct its associated misconceptions. Moreover, information on FGM/C health and legal consequences should be integrated within the medical school curriculum framing FGM/C within a wider sexual health discourse. This will help in changing the mindset of medical practitioners to see the long-term effects of FGM/C. A team of experts would be instrumental in ensuring that the topic is tackled from all different perspectives.

Health care providers need to be equipped with the appropriate counseling skills on FGM/C to be better able to convince clients to abandon the practice. The use of innovative methods in training and role plays with well-prepared scenarios of different customers’ requests of FGM/C and how to deal with each should be integrated in training sessions. Religious, moral and legal aspects of FGM/C need to be included in training sessions and in awareness raising activities to ensure the delivery of a holistic multi-dimensional message. Furthermore, the medical syndicate should take punitive measures against physicians who practice FGM/C by revoking their license.

### Study limitations and strengths

The study has some limitations. The study was conducted in 3 different governorates in an attempt to capture as much variability as possible. However, like any qualitative research the sample is not representative to the whole population. However, much strength exist in the study as it is the first study in Egypt highlighting the medicalization of FGM/C from perspective of both supply and demand side in various geographic areas (Upper Egypt, Lower Egypt, and Metropolitan Cairo) of Egypt that show differences in the practice. The “mystery clients” approach illustrated the real life responses of physicians regarding FGM/C performance.

## Conclusions

As suggested in the study findings, there remains to be a wide diversity of opinions regarding the medicalization of FGM/C in Egypt. This creates a culture of hesitation and further uncertainty which may motivate more mothers to seek a healthcare professional’s advice on cutting her daughter. It may thus cause a further rise in medicalization as long as social, moral and legal norms are not harmonized.

To the best of our knowledge, this is the first study to explore the reasons related to the involvement of healthcare professionals in the medicalization of FGM/C in Egypt from a qualitative perspective. However, the magnitude of the practice of FGM/C in health care providers’ families should be a focus of research, as they can never be agents of change if they continue the practice in their own families. Further research is also needed to document the number of health care providers who perform the practice. However, such a suggestion may be challenging given that FGM/C is criminalized in Egypt and the practice is being driven underground. Nevertheless, the use of technology and innovative methodologies may assist in extracting such data which in return will allow for better policy and programmatic responses to the issue of medicalization, and FGM/C in general.

## Additional file

Additional file 1: The Focus Group Discussion and In-depth Interview Guides file includes the English language version of the guides that were designed specifically for the purpose of this study and were tailored for mothers as well as nurses and doctors to understand their perceptions pertaining to the medicalization of FGM/C. (DOCX 18 kb)

## Data Availability

The datasets used and/or analyzed during the current study are available from the corresponding author on reasonable request.

## Abbreviations

EDHS: Egypt demographic and health survey
FGCS: Female genital cosmetic surgery
FGD: Focus group discussion
FGM/C: Female genital mutilation/cutting
HCP: Healthcare provider
IDIs: In-depth interviews
IRB: Institutional review board
LE: Egyptian pounds
MoHP: Ministry of health and population
NGO: Non-governmental organization
UNICEF: United nations children’s fund
WHO: World Health Organization
