# Supplementary material for: Health care providers’ and mothers’ perceptions about the medicalization of female genital mutilation or cutting in Egypt: a cross-sectional qualitative study
Source: BMC Int Health Hum Rights. 2019 Aug 27;19:26. doi: 10.1186/s12914-019-0202-x (PMC6712689; doi:10.1186/s12914-019-0202-x)
Supplement: Supplementary file 1 — The Focus Group Discussion and In-depth Interview Guides file includes the English language version of the guides that were designed specifically for the purpose of this study and were tailored for mothers as well as nurses and doctors to understand their perceptions pertaining to the medicalization of FGM/C. (DOCX 18 kb) [file 12914_2019_202_MOESM1_ESM.docx]

**THE FOCUS GROUP DISCUSSION AND IN-DEPTH INTERVIEW GUIDES**

***Assiut University-Faculty of Medicine- Department of Public Health***

**FOCUS GROUP GUIDE AND SCRIPT FOR DOCTORS AND NURSES / NEW RESEARCH PROJECT**

**Title of Project**: Medicalization of FGM/C in Egypt: Provider and Mother’s Perspectives

**Welcome Statement**

Welcome. The purpose of this meeting is to help researchers at the University of Assiut understand the drives of physicians/nurses practicing FGM/C in supporting the practice. Our approach is different than earlier studies by looking at FGM/C in the context of female sexuality. We are especially interested in how physicians and nurses understand gender, female sexuality, sexual health and FGM/C. This would help us to recommend changes in the medical and nursing education curricula on FGM/C. We are also interested in families and mothers’ perspectives as well. This will be dealt with in separate sessions.

We are audio taping this meeting for analysis later. This is with your permission. We destroy the tapes after transcribing the session. I would also like to introduce my research assistant………………….. who helps me recording this session.

I anticipate that we would take around 60-90 minutes to discuss issues. There will be some sensitive topics when we discuss female sexuality or sexual health. If you don’t want to participate, you are free to leave. For those uncomfortable participating in a focus group, we intend to invite them to an in-depth interview. There are no right or wrong answers to our questions. Openness and honesty are very important. Please note that it is strictly not allowed to share any information discussed during this focus group with anyone outside.

**Open Ended**

Are there questions about the study?

**Introductions**

Now that you know about our study, let’s find out a little bit more about you. Let’s go around the room and introduce ourselves. Please provide your first name only or a fake name. In a separate sheet, you will be asked to identify your age, year of graduation from medical school; marital status; have daughters and what age, work in urban or rural area, origins rural/urban etc.

*[This is to document that focus group constitution is adequate]*

**General views on FGM/C**

- How important is the issue of FGM/C to doctors in general? If you/other doctors would be asked to prioritize the health problems of Egypt on a list of 1 to 10, where would FGM/C be placed?
- Do many physicians approve of FGM/C or disapprove it? Why?
- Do many physicians practice FGM/C? Why?
- What do doctors think are the advantages and disadvantages of FGM/C?

**Knowledge of physicians on FGM/C**

- How informed are physicians on the levels of FGM/C in Egypt lately? And what are the current levels of the practice among girls? History of abandonment activities in Egypt? The shift of families to medical professionals to cut their daughters? How did this happen?
- Is it generally believed amongst physicians that FGM/C is religiously required?
- Some doctors say that some girls need to be circumcised. How have they learned about that? How do they differentiate between who needs and who doesn’t need to be circumcised?

**Community’s views on FGM/C and discipline**

- Do people in your community still practice FGM/C? How prevalent is it?
- Why was FGM/C generally associated with being ‘a proper girl’?

**FGM/C and the social network**

- To whom of your professional colleagues do you consult on professional matters? Mention the five most important to you (Number them only and say their relationship to you- No names)? Who has the greatest influence on your decisions?
- Would you discuss an issue like FGM/C with them? Or consult them?
- Do you think they would approve the practice of FGM/C? or not?
- Do you think they would practice FGM/C?
- On the issues of FGM/C would you consult anyone else other than your close network of professional friends? Who are they if any?
- Do you think they approve of criminalizing FGM/C and penalizing practicing doctors?

**FGM/C and sexual health**

- Regarding sexual health, how common are sexual health problems in Egypt?
- When men and women suffer from sexual health problems (other than infections), what is usually the first reason/thing they mention?
- Would men/women discuss sexual problems with their close network of friends? Why/why not?
- At which year in medical/nursing school do physicians learn about sexual health? And what do they learn exactly?
- What do the terms ‘sexuality’ and ‘sexual health’ and gender norms mean to physicians?
- How easy would a physician generally feel about consulting men/women on sexual health issues?

**Views towards anti-FGM/C law**

- Lately, there has been a doctor convicted because of practicing FGM/C? How physicians feel about that? Approve or don’t approve? Is this just? Or not?
- Is it right to have a law criminalizing FGM/C? Why yes? Why no?

**Assiut University-Faculty of Medicine- Department of Public Health**

**FOCUS GROUP GUIDE AND SCRIPT FOR MOTHERS / NEW RESEARCH PROJECT**

**Welcome Statement**

Welcome. The purpose of this meeting is to help researchers at the University of Assiut understand more about a practice that is still common in our society; namely ‘female genital mutilation/cutting’. We want to understand more on how mothers/primary care givers decide on circumcising their daughters and choosing the person to do the circumcision whether it is a nurse, a doctor or a daya (traditional birth attendant) in the community. What are the characters they seek in the provider? Who influences this decision? We will also play a game in which everyone will list the role of the persons they trust in daily child care issues (no names)? The five most important and who is the most influential in this group? Strength or trust?

We are audio taping this meeting for analysis later. This is with your permission. We destroy the tapes after transcribing the session. I would also like to introduce my research assistant………………….. who helps me recording this session.

I anticipate that we would take around 60-90 minutes to discuss issues. There are no right or wrong answers to our question, openness and honesty are very important. Please note that it is strictly not allowed to share any information discussed during this focus group with anyone outside.

**Open Ended**

Are there questions about the study?

**Introductions**

Now that you know about our study, let’s find out a little bit more about you. Let’s go around the room and introduce ourselves. Please provide your first name only or a fake name. In a separate sheet, you will be asked to identify your age, level of education, work status, number of daughters, residence, living arrangements, living with mother in law/not in the same house or building.

*[This is to document that focus group constitution is adequate]*

- Let us start by playing the game first: I would like to know who are the most important persons that you would consult on child care issues. I have placed a paper and a pencil for each one of you. I will demonstrate with one of you and then I and my assistant will go around to see who you list in this circle. You need only to write down their relationship to you (a colleague at work-sister in law/sister-mother- friend- neighbour etc.). We will use these markers to write + + + to each person to differentiate between those whose advise influences you mostly (strong =more ++++) (weak= + plus)
- Are there any others outside these groups you would consult on FGM/C? (Researcher to identify the weak links)
- Who do you live with? (N.B. Researcher needs to understand the living arrangement especially if the mother resides with an extended family or is in the presence of a mother in law)
- What is the level of approval/disapproval of the practice of FGM/C within the family?
- Does your family practice FGM/C currently? Or was it only in the past?
- What is the level of approval/disapproval of FGM/C among your network that you listed earlier?
- Do you believe that your trusted group would circumcise their daughters?
- Just go back in time and remember when … (ex. your last daughter was circumcised), can you tell me the story? How did you decide and were others involved in the decision-making? What made you circumcise her? Are there any ‘marking event’ that you noticed in the child which made you believe that the time has come?
- Who decides in the family who to go to for the procedure (practitioner)? What qualities do people seek in a FGM/C provider?
- What about your network? Who would they go to cut their daughters?
- Would they ever consult a doctor of FGM/C? Why?
- What would they do if the doctor advises them not to cut the girls?
- Do they approve of a legal punishment for doctors who perform FGM/C?
- What are the advantages and disadvantages of FGM/C?
- Have you thought to take a doctor’s advice on FGM/C? Why?
- If yes, have visited a doctor for that purpose? What did he say?
- What would you do if a doctor advises you against FGM/C?
- Were you exposed to anti-FGM/C messages? When and where?
- Is FGM/C generally believed to be a religious obligation? Probe: How did you learn about that and by whom? Do you know what is cited in the Hadieth and Quran making it an obligation?
- What do you know about the law in relation to FGM/C? (Probe also among the reference group)
- How much does it cost to circumcise a girl? (Mention the type of practitioner)

**Assiut University-Faculty of Medicine- Department of Public Health**

**IN DEPTH INTERVIEWS GUIDING QUESTIONS FOR DOCTORS AND NURSES**

**Welcome Statement**

Welcome. The purpose of this in-depth interview is to help researchers at the University of Assiut understand the drives of physicians/nurses practicing FGM/C in supporting the practice. Our approach is different than earlier studies by looking at FGM/C in the context of female sexuality. We are especially interested in how physicians and nurses understand gender, female sexuality, sexual health and FGM/C. This would help us to recommend changes in the medical and nursing education curricula on FGM/C. We are also interested in families and mothers’ perspectives as well. This will be dealt with in separate sessions.

We are audio taping this IDI for analysis later. This is with your permission. We destroy the tapes after transcribing the session. I would also like to introduce my research assistant………………….. who helps me recording this session.

I anticipate that we would take around 45 minutes to discuss issues. There will be some sensitive topics when we discuss female sexuality or sexual health. If you don’t want to participate, you are free to leave. There are no right or wrong answers to our questions. Openness and honesty are very important.

**Open Ended**

Are there questions about the study?

**Introductions**

Now that you know about our study, let’s find out a little bit more about you. Please provide your first name only or a fake name. In a separate sheet, you will be asked to identify your age, year of graduation from medical school; marital status; have daughters and what age, work in urban or rural area, origins rural/urban etc.

I would like to thank you for volunteering to elaborate more on FGM/C and how is how is it associated with female sexuality and gender norms.

**Physician’s personal views on FGM/C**

- How important is the issue of FGM/C to you as a doctor? If you were asked to prioritize the health problems of Egypt on a list of 1 to 10, where would you place FGM/C?
- Do you approve FGM/C or disapprove it? Why?
- Do you practice FGM/C? Why?
- (If physician has a daughter) Do you plan on circumcising your daughter? Why?
- What are the advantages and disadvantages of FGM/C in your opinion?

**Physician’s knowledge on FGM/C**

- Have you been informed on the levels of FGM/C in Egypt lately form the 2014 EDHS, by your organization? And what are the current levels of the practice among girls? History of abandonment activities in Egypt? The shift of families to medical professionals to cut their daughters? How did this happen?
- What about religion and FGM/C? Do you believe it is required by religion?
- Some doctors say that some girls need to be circumcised. Do you believe in this? How have they learned about that? How do they differentiate between who needs and who don’t need to be circumcised?

**Community’s views on FGM/C and discipline**

- In your family (larger family), how often is FGM/C currently practiced or was practiced in the past? Why?
- Why do you think was FGM/C associated with being ‘proper girl or wife’?

**FGM/C within the social network**

- To whom of your professional colleagues do you consult on professional matters? Mention the five most important to you (Rank them only by numbers- No names)? Who has the greatest influence on your decisions?
- Would you discuss an issue like FGM/C with them? Or consult them?
- Do you think they would approve the practice of FGM/C? or not?
- Do you think they would practice FGM/C?
- On the issues of FGM/C, would you consult anyone else other than your close network of professional friends? Who are they if any?
- Do you think they approve criminalizing FGM/C and penalizing practicing doctors?
- What about sexual rights and FGM/C? What is the relation between them?

**FGM/C and sexual satisfaction**

- What about FGM/C and sexual satisfaction among married couples? Do you think there is a relation between them? Why?
- Do you think pornography has any associations with supporting the practice? Why?

**FGM/C and sexual health**

- Regarding sexual health, how frequent do you encounter patients with sexual health problems in your clinical practice? Do you manage their complaints? Or do you refer them to someone else? Why?
- When men and women suffer from sexual health problems (other than infections)? What is the first reason/thing they mention?
- Would men/women discuss sexual problems with their close network of friends? Why?
- What type of complaints do you most often encounter? To which dimension of sexual health do they belong?
- We will discuss also what men think is ‘appropriate’ for women to express their sexuality within the context of marriage. Do you think this is related to FGM/C?
- At which year in medical school do you learn about sexual health? Have you been exposed to any counselling on sexual health issues of patients or couples?
- What do the terms ‘sexuality’ and ‘sexual health’ and gender norms mean to you?
- How easy would you feel to consult men/women on sexual health issues?
- How would you describe what you learned about sexual health, sexuality, gender, and FGM/C in medical/nursing schools in terms of content, and counselling skills?

**Physician’s views towards anti-FGM/C law**

- Lately, there has been a doctor convicted because of practicing FGM/C? How do you feel about that? Approve or don’t approve? Is this just? Or not?
- Is it right to have a law criminalizing FGM/C? Why yes? Why no?
